# HDX-Analyzer: a novel package for statistical analysis of protein structure dynamics

**DOI:** 10.1186/1471-2105-12-S1-S43

**Published:** 2011-02-15

**Authors:** Sanmin Liu, Lantao Liu, Ugur Uzuner, Xin Zhou, Manxi Gu, Weibing Shi, Yixiang Zhang, Susie Y Dai, Joshua S Yuan

**Affiliations:** 1 Institute for Plant Genomics and Biotechnology, Texas A&M University, College Station, TX, 77843, USA; 2 Department of Veterinary Pathobiolgy, Texas A&M University, College Station, TX, 77843, USA; 3 Department of Computer Science and Engineering, Texas A&M University, College Station, TX, 77843, USA; 4 Department of Plant Pathology and Microbiology, Texas A&M University, College Station, TX, 77843, USA; 5 Department of Statistics, Texas A&M University, College Station, TX, 77843, USA; 6 Office of the Texas State Chemist, Texas A&M University, College Station, TX, 77843, USA

## Abstract

**Background:**

HDX mass spectrometry is a powerful platform to probe protein structure dynamics during ligand binding, protein folding, enzyme catalysis, and such. HDX mass spectrometry analysis derives the protein structure dynamics based on the mass increase of a protein of which the backbone protons exchanged with solvent deuterium. Coupled with enzyme digestion and MS/MS analysis, HDX mass spectrometry can be used to study the regional dynamics of protein based on the m/z value or percentage of deuterium incorporation for the digested peptides in the HDX experiments. Various software packages have been developed to analyze HDX mass spectrometry data. Despite the progresses, proper and explicit statistical treatment is still lacking in most of the current HDX mass spectrometry software. In order to address this issue, we have developed the HDXanalyzer for the statistical analysis of HDX mass spectrometry data using R, Python, and RPY2.

**Implementation and results:**

HDXanalyzer package contains three major modules, the data processing module, the statistical analysis module, and the user interface. RPY2 is employed to enable the connection of these three components, where the data processing module is implemented using Python and the statistical analysis module is implemented with R. RPY2 creates a low-level interface for R and allows the effective integration of statistical module for data processing. The data processing module generates the centroid for the peptides in form of m/z value, and the differences of centroids between the peptides derived from *apo* and ligand-bound protein allow us to evaluate whether the regions have significant changes in structure dynamics or not. Another option of the software is to calculate the deuterium incorporation rate for the comparison. The two types of statistical analyses are Paired Student’s t-test and the linear combination of the intercept for multiple regression and ANCOVA model. The user interface is implemented with wxpython to facilitate the data visualization in graphs and the statistical analysis output presentation. In order to evaluate the software, a previously published xylanase HDX mass spectrometry analysis dataset is processed and presented. The results from the different statistical analysis methods are compared and shown to be similar. The statistical analysis results are overlaid with the three dimensional structure of the protein to highlight the regional structure dynamics changes in the xylanase enzyme.

**Conclusion:**

Statistical analysis provides crucial evaluation of whether a protein region is significantly protected or unprotected during the HDX mass spectrometry studies. Although there are several other available software programs to process HDX experimental data, HDXanalyzer is the first software program to offer multiple statistical methods to evaluate the changes in protein structure dynamics based on HDX mass spectrometry analysis. Moreover, the statistical analysis can be carried out for both m/z value and deuterium incorporation rate. In addition, the software package can be used for the data generated from a wide range of mass spectrometry instruments.

## Background

Protein intrinsic dynamics has been more and more recognized as an important consideration for protein functions [1]. Several recent studies have revealed that protein dynamics plays essential roles in the catalysis and other functions [1,2]. Among the different techniques, HDX mass spectrometry stands out as a relatively high throughput platform to probe the backbone dynamics of the proteins [3-6]. HDX mass spectrometry has been broadly applied to study protein dynamics and structure, particularly for the protein binding with ligands, substrates, DNA and other molecules [4,7-10]. Such analysis has enabled the illustration of mechanisms for enzyme substrate interaction and the molecular determinants during protein binding [11,12]. The fundamental concept of HDX mass spectrometry analysis is based on the mass increase of a protein when the protein protons exchanged with solvent deuterium [6]. The rate and percentage of the H/D exchange can be measured by mass to charge ratio (m/z) of the protein. The HDX mass spectrometry can be utilized to study both the global and regional protein conformational changes [13,14]. Coupled with protein digestion and chromatography separation, the HDX mass spectrometry enables characterizing different regions of protein for H/D exchange based on the peptide H/D exchange rate or the m/z of the peptides. In a differential HDX experiment, typically two protein forms will be compared. The *apo* protein and the ligand bound protein are subjected to HDX experiment in a parallel mode. The information allows one to understand which region of the protein is more stabilized or destabilized upon ligand binding in the solvent exchange reaction [3-5,14-22]. If more H/D exchange is observed in a particular region, the protein region is more dynamic in the solvent exchange reaction, meaning that the region is more flexible or less stable in HDX. In a typical differential HDX experiments, the protein of interest is subject to different exchange times in its *apo* form and protein ligand complex with technical replicates. The data processing for HDX mass spectrometry thus requires us to compare a large set of the m/z values or percentages of deuterium incorporation for the same peptides derived from *apo* protein and ligand bound protein.

Various software platforms have been developed to analyze the HDX data. Among them include HX-Express, Deuterator, HD Desktop, DEX, Hydra, TOF2H etc. Most of these HDX data analysis software packages focus on calculating the m/z value from the MS raw data for the deuterated peptide, and then evaluate the m/z value increase according to time. For example, HX-Express is a semi-automated software package which exports deuterium uptake curve and peak width plots based on Microsoft excel application [23]. Compared with HX-Express, Deuterator is more automated and can deconvolute overlapping mass peaks. Meanwhile, Deuterator acts as a web-based server to process HDX data sets on line [24]. Furthermore, HD Desktop is built on top of deuterator, and integrates more tools for data extraction displaying visualization components [25]. DEX uses a Fourier deconvolution method for computing high-resolution mass spectrometry data [26]. Hydra executes through a user-defined workflow, by which deuterium incorporation values are extracted and visualized in tabular and graphical formats. Hydra also automates the extraction and visualization of deuterium distribution values for large data sets [27]. TOF2H focuses on interpreting MALDI-based HDX data and also builds up a pipeline for automated data processing [28]. Despite the significant progresses mentioned above, most software uses absolute differences between HD exchange rates as an evaluation of the differential structure dynamics changes, whereas few tools enables statistical evaluation of differential HD exchange among different conditions. It is noted that CalcDeut [29] evaluate the statistical distribution of deuterium incorporated into protease digested peptide fragments to compensate data truncation due to instrument signal to noise ratio. Hydra provides a prototype of student t-test evaluation and calculates a p-value for the differential HD exchange. Despite the significant advances made by Hydra, due to the inherent limitation of regular Student’s t-test, the statistical treatment provided by Hydra is suitable to analyze data at one exchange time point, though HDX experiments always involve multiple time points and the HDX rate is time-dependent. In order to address the issue, we integrated the pairwise t-test, multiple regression and ANCOVA models to provide the accurate evaluation of the differential HD exchange for proteins under different treatments. It needs to be realized that the HDX exchange of a peptide is time-dependent, and the usage of regular Student’s t-test to compare the same peptide throughout the whole HDX time course is not accurate. A pairwise t-test can be used. A multiple regression model or ANCOVA model is more suited for this scenario. The statistical analysis of differential exchange rate for peptide is crucial for evaluating if significant differential structural dynamics changes exist for a specific peptide region or not. In many studies, a large difference in exchange rate may not reflect the differential structure dynamics changes if the standard deviation for the exchange rate is high. For this reason, we have developed a new platform offering various choices for statistical evaluation of HD exchange rate among different samples.

The integration of statistical analysis with data processing is challenging. In terms of statistical analysis, several software environment including SAS, SPSS, and R can be employed. Among these packages, R is the open source program and can be easily obtained from internet free of charge. Despite the various advantages of R, the software environment does not have strong user-interface supports and thus requires certain level of expertise. In order to develop user-friendly statistical software for HDX mass spectrometry analysis, we hereby employ the latest RPY2 package to connect the statistical module of R with a data processing module implemented by Python and a user-interface implemented by wxpython. Many programming languages including C, java, Perl, Python can be used for data processing and UI development, and each programming language has its pros and cons. Among these programming languages, Python is chosen as the main developing language for two reasons: First, the existed RPY package allowed the seamless and effective implementation between the data processing module and statistical module in R. Second, various BioPython packages have been developed for the analysis of biological data which allows fast and easy embedding. For these reasons, we have developed the HDX mass spectrometry analysis software HDXanalyzer using Python, R, and RPY2 packages.

In this article, we hereby present a novel software package HDXanalyzer for statistical analysis of HDX mass spectrometry data to evaluate the protein structure dynamics changes. The software package includes three major components, the data feeding and processing module, the user interface, and the statistical analysis module. The data processing module is developed in Python to process a batch of excel input files containing the m/z value of the peptides from different experiments. The pre-formatted m/z value of the peptides will be processed to derive the centroid of the peptide peaks or the percentage of deuterium incorporation for the statistical analysis. The data is then processed by the Figure Generator to create graphs to visualize the differential HD exchange rates in *apo* and ligand bound protein. Further statistical analysis is carried out by R, where two statistical methods are used. The Paired Student’s t-test is used to compare either the centroid values of the m/z value or the deuterium incorporation rate to derive point estimation, confidence intervals, and p value to indicate if significant differences in structure dynamics exist or not. In addition, the multiple regression (or ANCOVA) model is also involved for the similar analysis through linear combination of the intercepts. HDXanalyzer thus provided novel solutions toward ultimate quantification and statistical evaluation of the structure dynamics changes in the HDX mass spectrometry experiments. The software package addressed the imminent need of statistical evaluation for the HDX mass spectrometry analysis and can be expanded to other applications for HD exchange studies by other techniques.

## Implementation

### I. Data processing as implemented by python

**Figure 1:**
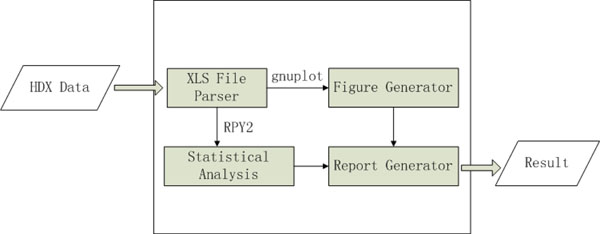
The implementation flow of the HDXanalyzer.

As shown in Figure 1, HDXanalyzer includes several modules, data processor, statistical analysis module, and user interface. HDX mass spectrometry usually involves two types of data, the MS/MS analysis for peptide sequence identification and the MS analysis for m/z value of the peptide peaks in different protein formats and statuses (i.e. *apo* form, ligand bound form, proteins that has been subjected to different HDX exchange time points). The MS/MS analysis often allows us to correlate the peptide ID with a sequence, which is beyond the scope of HDXanalyzer. HDXanalyzer is mainly dealing with the MS analysis data. The raw MS data was exported for intensity and m/z values in the excel format for each peptide as shown in the online Additional Files available at http://people.tamu.edu/~syuan/hdxanalyzer. The Additional Files contain the sample input data derived from HDX mass spectrometry analysis of xylanase enzyme and the executable file for HDXanalyzer software. The data is in a compressed package available at the aforementioned link. The HDXanalyzer then takes all the pre-formatted excel file containing peptide ID and m/z values of the peptides at different HDX exchange time points as the input. A python package for reading and formatting information from excel files named as xlrd was used to develop a parser to extract and process excel spreedsheets. The parser allows us to extract all m/z values and intensity of the peptides. The peptide ID and the treatment (*apo* or ligand bound) will also be extracted.

The data processing can derive two types of variables for the statistical analysis, centroid of the peptide in form of m/z value or deuterium incorporation rate. Centroid values of each peptide are derived based on the m/z value of the peaks generated by MS analysis. For deuterium incorporation rate, the weighted average m/z values of each peptide ion isotopic cluster are calculated. Basically, the deuterium incorporation rate is calculated based on the centroid of the peptide m/z value and is in form of percentage. The deuteration level of each peptide is calculated based on Equation 1, and corrections for back-exchange are made based on 70% deuterium recovery and accounting for 80% deuterium content in the ion-exchange buffer. These corrections can be defined by users in the data pre-processing procedure.

where m/z (P), m/z (N), and m/z (F) are the centroid values of partially deuterated peptide, nondeuterated peptide, and fully deuterated peptide, respectively [24].

The resulted data are then processed into a table format and loaded to Figure Generator to create visualization of the dynamic status of peptide at different time points. Specifically, the figures are graphs displaying the m/z value or deuterium incorporation rate of a peptide at different exchange time. Gnuplot, an open source GNU plotting tool under UNIX/linux, with counterpart in MSDOS & Windows system, was employed to implement the Figure Generator. The advantages of Gnuplot lies in two aspects including the availability from either GNU projects or internet free of charge, as well as the convenience of automated generating multiple outputs using its corresponding scripting language. Besides the graphic display of the HDX data, statistical analysis is carried out to generate the point estimation for differential m/z value or incorporation rate, the confidence intervals and the p value.

### II. Statistical models and implementation

Statistical analysis is employed to evaluate if a peptide or a region of the protein has significant changes in structure dynamics or not. Such changes are reflected in the differences of either centroid m/z values or the deuterium incorporation rates during the HDX experiments. The m/z value or deuterium incorporation rate from different peptides can be compared with different statistical models to derive parameter estimation and p value. The parameter estimations allow us to evaluate the levels and variations of the differences in structure dynamics of a protein region, and the p value allows us to determine if the differences are significant or not.

Two types of statistical models are used. First, a Paired Student’s t-test (Pairwise t-test) is used to compare the m/z value or percentage of deuterium incorporation for peptides from *apo* or ligand binding proteins. Paired Student’s T-test is used instead of the regular T-test because of the time effects in the HDX experiments. More specifically, the m/z values or the percentages of deuterium incorporation for a peptide will increase as the hydrogen deuterium exchange time gets longer. As these two values are time dependent, both will reach a plateau when the exchange time is long enough. For this reason, the Paired t-test is used to avoid the time point effects. Besides the pairwise t-test, the multiple regression model or ANCOVA model are utilized to compare the m/z or incorporation rate differences between peptides from the two types of proteins (*apo* and ligand bound). The multiple regression model is as shown in Equation 2. The linear combination of the Group effects allows us to compare the differences between *apo* and ligand-bound proteins. For either model, the point estimation of mean differences, confidence intervals, and p value will be computed.

*Y* = *β_T_X_Time_* + *β_G_X_Group_* + β_TG_X_Time_*X_Group_ Equation 2

where Y is the dependent variable that can be either the m/z value or the deuterium incorporation rates of different peptides. Y is dependent on the effects of time points and different groups from either *apo* or ligand bound proteins. The combination of the two effects may also influence the dependent variable.

### III. RPY for integrating the different components

The integration of statistical analysis, data processing, and visualization is usually challenging. The recent developed RPY allows us to integrate the statistical feature of R and the user interface as well as the data processing features of Python. As an open-source language, R has the unique advantages over other statistical languages for software development. RPY enables us to employ the R for statistical analysis of HDX mass spectrometry data. We have also used RPY2 to provide a low-level interface to R. The Python-based system thus can directly call R function through RPY and the software efficiency and effectiveness are greatly improved.

### IV. User interface as implemented by wxpython

**Figure 2:**
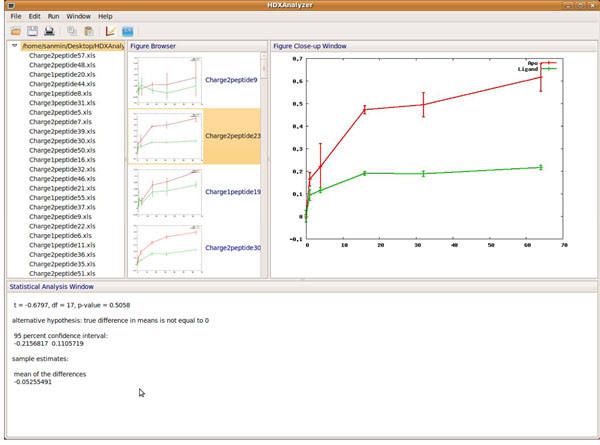
The overview of the user interface.

The user interface of HDXanalyzer is developed with Wxpython as shown in Figure 1 and 2. Wxpython is a Python extension model, which works as a wrapper for the cross-platform GUI API wxWidgets for the Python programming language. We have developed the user interface including a menu bar, a tool bar, and four windows. The four windows are data manager window, figure browser, enlarged figure, and statistical analysis windows. Data manager window shows the spreadsheets (or the peptides) for the data analysis. Figure browser window shows a list of the graphs for comparing the m/z value or deuterium incorporation rate for all peptides listed in the data manager window. All of the graphs are clickable and can be viewed in the enlarged figure window. The statistical analysis window displays the statistical analysis results.

## Result and discussion

HDXanalyzer is implemented as a software package to enable the statistical analysis of HDX mass spectrometry data and to allow the evaluation of protein structure dynamics changes. In order to demonstrate the software application, we first analyze a previously published dataset for the HDX mass spectrometry analysis of xylanase enzyme. The example data is available in the online supplementary document. Furthermore, we also apply HDXanalyzer to analyze two peptides from a recent publication, where the HD exchange for the two peptides were statistically evaluated. We hereby discuss the usage of the software, present the output, compare the different results from different statistical models, and interpret the results.

### I. The input data format and the usage of the package

The HDXanalyzer aims to integrate statistical analysis for comparing structure dynamics of protein upon ligand or substrate binding. As discussed in the Implementation section, the software takes a batch of pre-formatted excel files containing m/z values for multiple peptides of different treatment and time points as shown in Supplementary File 1 available online (the HDX_Xylohexaose.rar dataset). The data pre-formatting will allow the software to process a uniform input of HDX mass spec data from different instruments. The sample input file is derived from a xylanase structure dynamics study and the m/z values of the peak area for the peptides are included. Each input excel file will contain several sheets for the data from different peptides and treatments. The spreadsheet contains peptide ID, m/z value, charge state, time points for deuterium treatment, and the ligand name to separate different experimental sets, e.g., *apo* set and ligand set. The peptide ID can be corresponding to a certain peptide sequence. The upload function is available from the user interface, where input file can be read and processed to generate m/z centroids and deuterium incorporation rates of the peptides as aforementioned. The data are therefore further analyzed for visualization and statistical analysis.

### II. The output of HDXanalyzer

**Figure 3:**
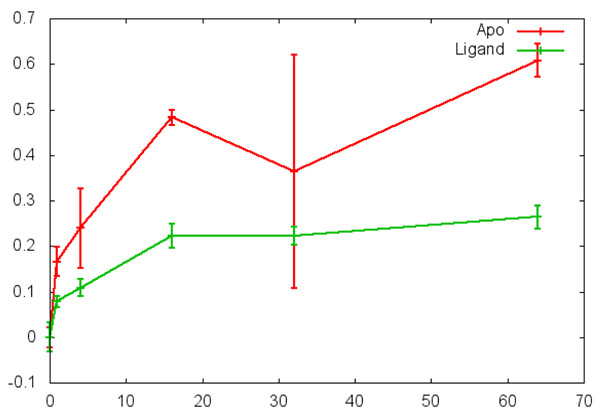
The enlarged figure of a graph for comparing the sample peptide from *apo* and ligand-bound protein.

**Figure 4:**
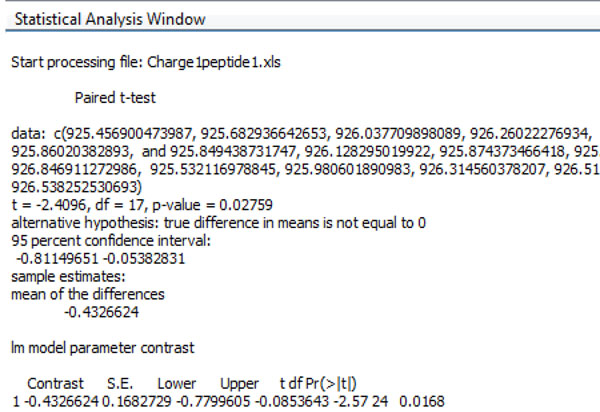
The output for the statistical analysis window.

The data output includes three parts as shown in Figure 2. The upper left panel is the data manager window and contains the peptide list. The upper middle panel is the figure browser window and contains the graphs to compare the trends of HD exchange for all the peptides from the input file. In each graph, the X axis is the time after the deuterium incubation, and the Y axis is either the m/z value of the centroid or deuterium incorporation rate. The user can choose the output in either format. The same peptide from the *apo* protein and the ligand-bound protein are marked with different colors in the graph. Each graph in the middle panel is clickable, and the enlarged graph for each peptide is presented in the enlarged figure window in the upper right panel as shown in Figure 2 and 3. When the graph is clicked, the statistical analysis can be executed for the particular peptide and the output is presented in the statistical analysis window at the bottom (Figure 4). The statistical analysis output is typical R output with the point and confidence interval estimation as well as the p value. The parameter and p value estimation can be generated for either centroid of the peptide or the deuterium incorporation percentage. Statistical analysis can be executed for all the peptides and the output can be presented together in the statistical analysis window.

### III. The interpretation and comparison of different statistical models

As aforementioned, two types of statistical analyses are implemented for HDXanalyzer. Both Paired Student’s T-test and the linear combination of intercept for group (*apo* vs. ligand) effects in multiple regression (ANCOVA) model are used to derive parameter and p value estimation. A very important decision for data processing is the choice of time point. The early time points after deuterium exchange, especially for exchange less than 1 minute, may lead to very limited exchange even in *apo* protein. In such case, the statistical analysis of data from these time points cannot represent the real deuterium incorporation level. Therefore, HDXanalyzer offers the users the option to choose the time points for the analysis and we have focused on the time points after ten minutes in our analysis of the example dataset.

**Figure 5:**
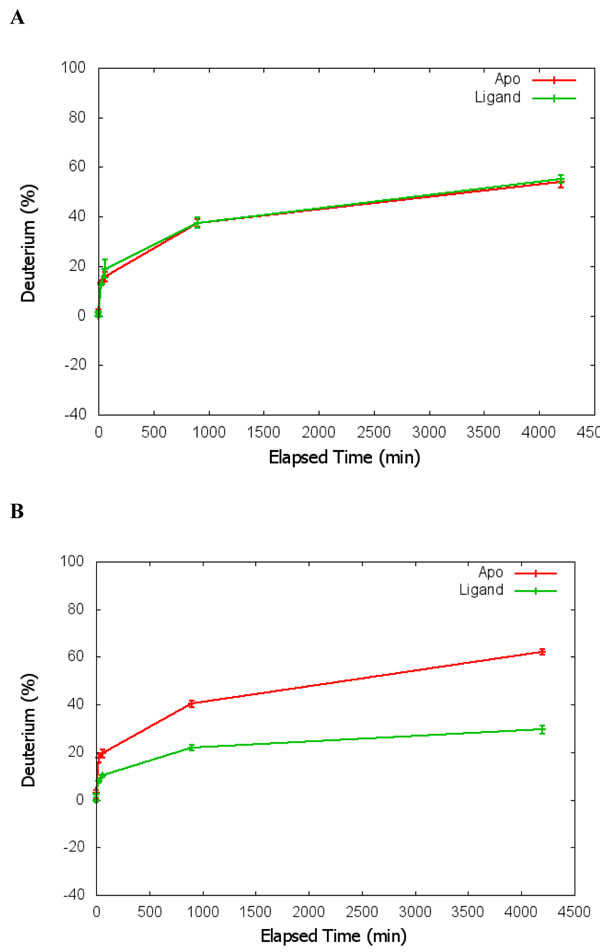
Verification of data analysis using previously published estrogen receptor data. A panel is a peptide showing no significant changes in HDX; and B panel shows a peptide with significant changes in HDX.

The data interpretation is another important consideration. Several key values are presented in the statistical analysis readouts. The p value presents the estimation of whether a region has significant differential structure dynamics or not. A small p value on either deuterium incorporation rate or centroid of m/z indicates that the peptide has significant differential structure dynamics changes between the *apo* and ligand interaction. The point estimation of the mean differences and the confidence intervals estimates the levels of the changes. Both parameter estimation and the p value are important in interpreting the HDX mass spectrometry data to render reliable conclusions. In addition, the statistical analysis results can present both the Paired Student’s t-test results and the multiple regression model. As shown in Table 1, the results of xylanase analysis from both types of statistical analysis correlate with one another. To further validate the analysis results, we also analyze the HDX data from another previously published dataset [30]. Figure 5 shows the results of statistical analysis for two peptides presented in Figure 1S B and C in the previous publication by Dai et al. [30]. As shown by Figure 5, the peptide in panel A did not show significant differential HDX, whilst the peptide in panel B shows significant differential HDX with P < 0.001. The results from HDXanalyzer correlates well with the previous statistical analysis carried out by regular statistical analysis software PRISM by Dai et al.[30]. The comparison highlighted that HDXanalyzer is reliable.

**Table 1:** The comparison of statistical analysis results from Paired Student’s t-test and multiple regression.

Analysis Type	Mean	LCL	UCL	p Value
Multiple Regression	0.4522	0.3389	0.5654	0
Paired t-test	0.4452	0.3393	0.5649	0
Multiple Regression	0.2362	0.1157	0.3567	0.0004
Paired t-test	0.2362	0.1237	0.3487	0

### IV. The adaptability to data from different instrument sources

Another strength of HDXanalyzer is that the data pre-processing allows the package to analyze HDX mass spectrometry data from a wider range of instruments. Most of the current software packages are developed on one type of instrument or another and are more adapted toward the high resolution data from very high-end instruments. In order to allow HDXanalyzer to process data from various instruments, we decide to handle the pre-processed data as shown in the supplementary file. The data pre-processing step will be able to handle the HDX mass spectrometry data from different instrument types and the pre-processed data can then be analyzed by HDXanalyzer regardless of instrument types.

### V. The overlay of 3D structure for differential structure dynamics of xylanase

**Figure 6:**
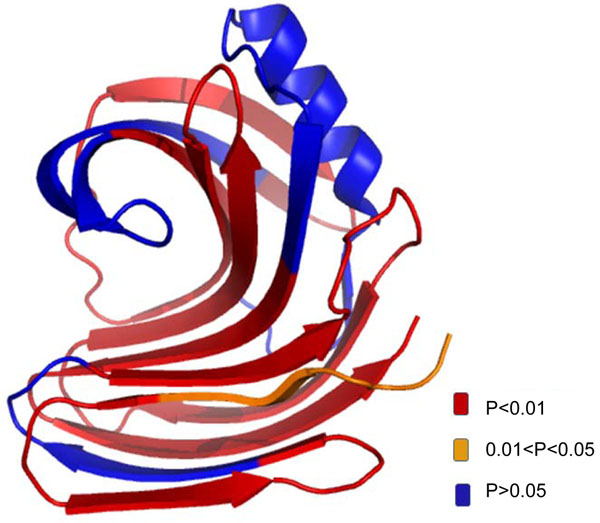
The overlay of p value with 3D structure of xylanase. The color legend indicats the level of confidence.

The statistical analysis allows us to identify which region of the protein has significant structure dynamics changes upon ligand binding. As shown in Figure 6, the substrate binding of xylanase has introduced significant structure dynamics changes in many different regions of the protein. The overlay of 3D structure and significance of the changes provided another way to interpret the HDX mass spectrometry data. For example, when we overlay the p value of the different peptides with the protein structure, the regions with significant changes are highlighted and the information can help to understand the enzyme catalysis mechanisms. HDXanalyzer thus provided a powerful tool for statistical analysis of structure dynamics data, which has not been achieved for previous HDX mass spectrometry analysis software.

## Conclusion

HDXanalyzer as a statistical analysis software has enabled the accurate evaluation of the changes of protein structure dynamics. The software integrates the graphic visualization and statistical analysis to enable the effective evaluation of the differential structure dynamics in the HDX mass spectrometry experiments.

## Authors' contribution

JSY and SYD oversight the work and designed software together with SL, LL, and XZ. XZ developed an early version of the software in python, and LL completed the development with most of the functions through imbedding R module for pairwise Student’s t-test. SL implemented RPY2 and both pairwise Student t-test and multiple regression (ANCOVA) model together with MG and JSY. SL greatly improved the software implementation and developed UI with wxpython. MG provided valuable suggestions for multiple regression model development. UU provided the pre-formatted data for the software development, valuable advises for implementation, and generated the overlaid the statistical results with 3D structure of the xylanase enzyme. WS and YZ provided valuable suggestions for the implementation. JSY and SYD drafted most parts of the article and finalized the draft. SL drafted most of the implementation and UU drafted part of the implementation. JSY, SYD and LL revised the article.

## Competing interests

The authors declare no competing interests.
